# Predicting the Risk of Decompensation in Patients With Cirrhosis: A Validated Machine Learning Approach

**DOI:** 10.14309/ajg.0000000000004004

**Published:** 2026-03-25

**Authors:** Philip J. Johnson, Anton Kalyuzhnyy, Arturas Grauslys, Hidenori Toyoda, Erica Villa, Omar Elshaarawy, Stephen Stewart, Ian A. Rowe, Jessica Shearer, Indra Neil Guha

**Affiliations:** 1 Department of Molecular and Clinical Cancer Medicine, University of Liverpool, Liverpool, United Kingdom;; 2 Computational Biology Facility, University of Liverpool, Liverpool, United Kingdom;; 3 Hangzhou Zhisuan Anneng MedTech Ltd., Hangzhou, China;; 4 Eliptica, London Cancer Hub, Sutton, London, United Kingdom;; 5 Department of Gastroenterology, Ogaki Municipal Hospital, Ogaki, Japan;; 6 CHIMOMO Department, University of Modena & Reggio Emilia, Modena, Italy;; 7 Department of Gastroenterology and Hepatology, University Hospitals of Liverpool, United Kingdom;; 8 Department of Gastroenterology and Hepatology, National Liver Institute, Menoufia University, Egypt;; 9 Department of Gastroenterology, The Mater Hospital, Dublin, Ireland;; 10 Leeds Liver Unit, St. James's University Hospital & Leeds Institute for Medical Research, University of Leeds, Leeds, United Kingdom;; 11 NIHR Nottingham Biomedical Research Centre, Nottingham University Hospitals NHS Trust and the University of Nottingham, Nottingham, United Kingdom.

**Keywords:** hepatic decompensation, cirrhosis, machine learning, risk stratification, chronic liver disease

## Abstract

**INTRODUCTION:**

There is an unmet need for simple tools to predict development of hepatic decompensation among patients with compensated cirrhosis. We applied machine learning to several international Data sets to develop and validate a straightforward predictive model of decompensation.

**METHODS:**

We used routinely available clinical and laboratory data from 575 patients with compensated cirrhosis from the training cohorts in Nottingham (United Kingdom) and Modena (Italy), with a median follow-up of 4 years. Based on these data, we developed a predictive model using a random forest classifier and validated it across independent international populations involving over 2,100 patients from Dublin (Ireland), Menoufia (Egypt), Leeds (UK), and Ogaki (Japan).

**RESULTS:**

In the training cohorts, 22% of patients developed liver decompensation. Using machine learning, we developed risk of decompensation in cirrhosis (RODIC), a well-calibrated model based on albumin, bilirubin, and the Fib-4 value (area under the receiver operating characteristic curve = 0.86; weighted F1 score = 0.82) which predicts the risk of decompensation within 3 years (access free of charge at: https://antonkaly.pythonanywhere.com/predict). RODIC showed strong performance across all validation sets, with area under the receiver operating characteristic curve scores ranging from 0.67 to 0.80 and weighted F1 scores from 0.70 to 0.81. Moreover, the model was effective regardless of cirrhosis etiology. For hepatitis C virus-positive patients, RODIC remained reliable irrespective of whether they achieved sustained virologic response.

**DISCUSSION:**

Our validated machine learning model based on readily available clinical, and laboratory features accurately quantitates the risk of liver decompensation in patients with compensated hepatic cirrhosis.

## Graphical abstract

**Figure:**
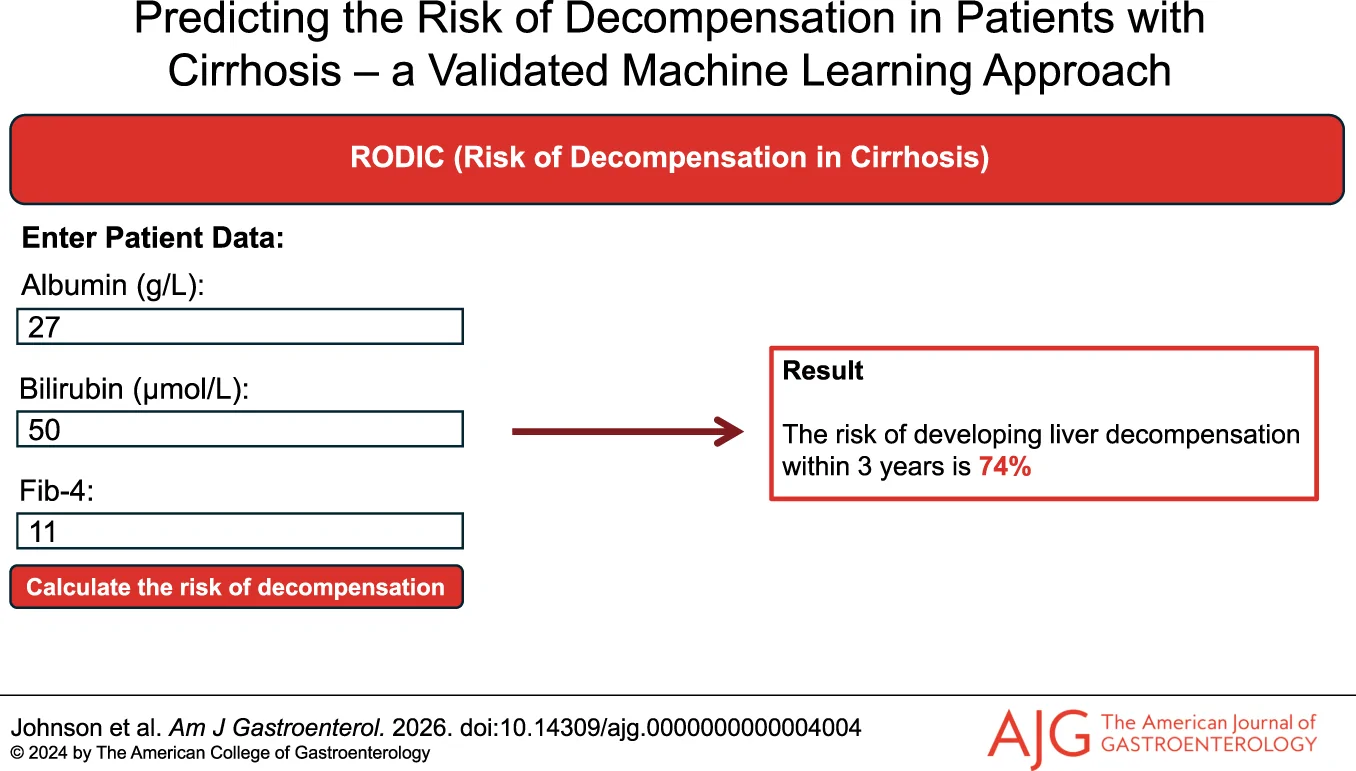

## INTRODUCTION

The major complications of hepatic cirrhosis namely, ascites, bleeding from gastroesophageal varices and encephalopathy consume significant resources across all countries and regions and are jointly referred to as decompensation events. A recent Baveno consensus statement (1) recognized that validation of circulating biomarkers for prediction of decompensation in all etiologies is an important part of the current research agenda. Direct assessment of portal vein pressure is an attractive candidate for prediction of decompensation, but measurement of hepatic vein portal gradient (HVPG) is invasive and logistically challenging (2). As a result, it has been acknowledged that simple yet effective tools for predicting decompensation represent an unmet need (2).

Successful risk-stratification would permit more rational distribution of resources in the management of decompensation. It has also been suggested that in patients with compensated cirrhosis it is more clinically relevant to seek predictors of decompensation rather than mortality, particularly if the risk of decompensation can be accurately assessed years in advance (3). Earlier studies have identified major clinical variables (4,5) associated with decompensation, the challenges faced by attempts to predict this event, and the limitations of existing models (2,6,7). We have previously proposed such a tool based on the albumin-bilirubin grade (ALBI) score (8), and an index of the degree of fibrosis (Fib-4) (9). Although the model could distinguish between those at a high risk and those at low risk, there were several limitations (2). Specifically, the model did not quantify the risk, calibration was not optimal, and the etiology of the cirrhosis was not considered. These issues are addressed in the present study.

The strengths and limitations of machine learning are being increasingly recognized and applied to clinical situations including chronic liver disease (10). We now use a formal supervised machine learning algorithm based on random forest (RF) classification (11) to enhance the accuracy of decompensation prediction. RF models are centered around building multiple decision trees for nonparametric classification and regression tasks. Each tree is constructed using a random subset of data through using bootstrapping with replacement. At each split within a tree, RF randomly selects a subset of patient features to determine the optimal way to separate the data into distinct classes by evaluating various thresholds. For predictions on new data, the input is passed through each trained tree within the forest, where the established splits and thresholds dictate the path taken, leading to a classification. The final class prediction is obtained by the majority vote after aggregating the classifications from all trees (11,12). Several online tools such as scikit-learn (13) also allow quantifying the risk of obtaining a specific classification. Furthermore, the RF algorithm allows to assess the importance of the involved patient features by calculating the reduction in node impurity (measured by the Gini index) across all trees (11) and by performing shapley additive explanations (SHAP) analysis which quantifies the magnitude and direction of each feature's contribution to the predicted risk (14).

Currently, the most widely used prognostic model is model for end-stage liver disease (MELD) (15). As the name implies, this model and its recent modifications (16) were designed to predict mortality among patients with decompensated (end-stage) cirrhosis. However, a recent large-scale examination of several models for predicting outcome of compensated cirrhosis found that although MELD was the best predictor of mortality/hospitalization, Fib-4-based models were optimal for predicting decompensation (17).

In this study, we aimed to quantitatively predict the risk of decompensation, enabling healthcare systems to efficiently allocate resources and identify cirrhotic patients at high risk of decompensation. Through applying the novel RF approach, we present a straightforward model that can be applied in a clinical setting to accurately predict the risk of decompensation within a period of 3 years.

## METHODS

The analysis in this study was performed in adherence with the Transparent Reporting of a multivariable prediction model for Individual Prognosis Or Diagnosis + artificial intelligence guidelines for the development and reporting of artificial intelligence-based prediction models (18).

### Patients

The cohorts used for training our model were the prospective, longitudinal Nottingham compensated cirrhosis cohort from the United Kingdom (UK) and the Modena cohort from Italy. The Nottingham cohort has been described in detail previously and updated to the year 2018 (9). Briefly, it comprised 181 patients with cirrhosis who had no present or previous evidence of decompensation or hepatocellular carcinoma (HCC). Patients were followed up at approximate intervals of 6 months from May 2010 to July 2018, with routine blood tests taken at each visit.

The Italian cohort comprised 545 patients with compensated chronic liver disease, recruited between 2010 and 2018 in the Gastroenterology Department of the University of Modena and Reggio Emilia. After excluding patients with previous episodes of decompensation, 394 were entered into the analysis with a median follow-up of 3.6 years at 5 years, and 5.6 years at 10 years. At the time of enrolment, no patients had evidence of HCC development (19).

We also had access to 4 international independent cohorts (Table 1) from Leeds (UK), Dublin (Ireland), Menoufia (Egypt) and Ogaki (Japan) which were used for validation. The former 3 validation cohorts have been described previously and updated (9). The Ogaki cohort was prospectively-accrued and consisted of 1,648 cirrhotic patients from the Ogaki Municipal Hospital who were undergoing surveillance for HCC.

**Table 1.**
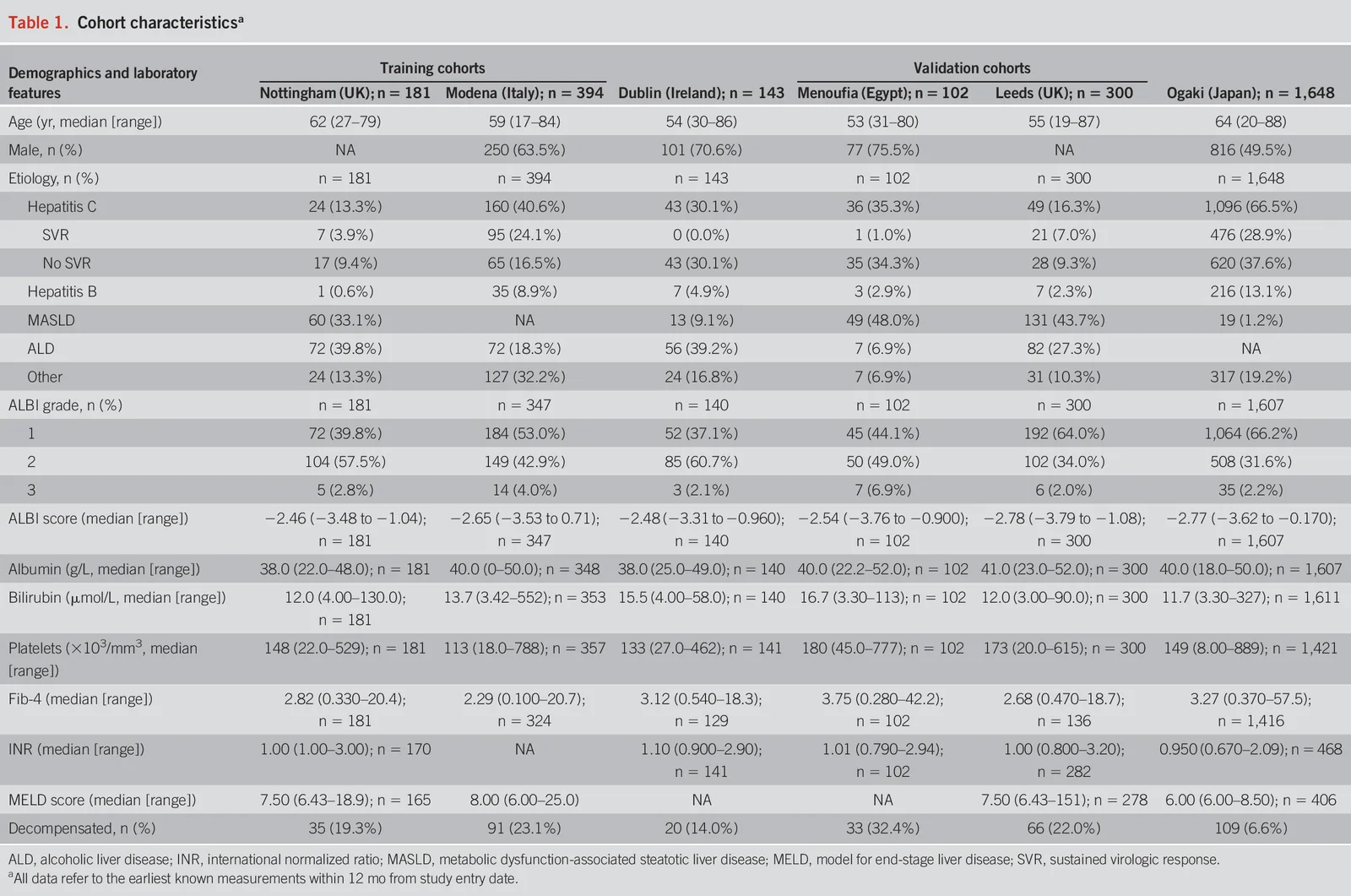
Cohort characteristics^a^

In all cohorts, cirrhosis was diagnosed by the treating clinician based on standard criteria, including imaging, histology, clinical features and evidence of portal hypertension. The presence of ascites, hepatic encephalopathy (HE) or variceal hemorrhage was confirmed by senior hepatologists in accordance with local practice.

### Model development

In total, 181 patients from the Nottingham cohort and 394 patients from the Modena cohort (Table 1) with clinical data were used to develop the RF model. In the Nottingham cohort, missing patient measurements were imputed by linear interpolation using the 4 nearest longitudinal values surrounding the missing measurement, provided these values were recorded within 1 year of the missing observation, as larger gaps were considered likely to reflect substantial changes in biomarker values that could not be reliably estimated. Overall, ∼11% of measurements could not be imputed (primarily because of having consecutive missing values in the early years of follow-up) and were therefore excluded from model development. On average, after imputation there were 14 complete measurements per patient in the Nottingham cohort. In the Modena cohort, we had access to a single assay from each patient recorded at study entry and in total, 315 patients had a complete set of biomarker measurements that were used to train the model. The specific details of model development, selecting and analyzing patient features, as well as model validation are available in Supplementary Material (see Supplementary Digital Content, http://links.lww.com/AJG/D916).

### Evaluation of model performance

The model's discriminatory power was assessed by the average area under the receiver operating characteristic curve (AUC-ROC) across 1,000 iterations, along with a weighted average F1 score which balances sensitivity (recall) and positive predictive value (precision) (20). The weighted F1 score accounts for class imbalance by considering the number of instances in each outcome class (decompensated and non-decompensated), thereby providing a comprehensive single-number summary of model performance. The weighted average F1 score was computed using the scikit-learn implementation (13), which calculates the F1 score for each class and averages them weighted by the number of true instances (support) in each class.

Calibration was evaluated using a standard calibration plot, mean absolute error and mean squared error, which measure the average difference between predicted and observed outcomes (13,21). To improve calibration of the resulting risk scores, we applied isotonic regression, a monotone, nonparametric method that adjusts predicted probabilities so that they better align with observed outcomes across the entire risk spectrum (22).

Although the RF model did not use the longitudinal nature of the Nottingham cohort data, it was used to examine changes in RODIC scores over time leading to decompensation. The predicted scores for every longitudinal measurement were averaged and smoothed using locally estimated scatterplot smoothing regression (23) and presented with 95% CIs. Where possible, an analogous locally estimated scatterplot smoothing analysis was performed for MELD, Fib-4, and ALBI scores.

## RESULTS

### Model performance and calibration

Out of 575 patients in the training cohorts, 126 (22%) progressed to liver decompensation during follow-up. Our resulting RF model, risk of decompensation in cirrhosis (RODIC) provided a risk of developing liver decompensation within 3 years from applying the model, based on patient's albumin, bilirubin, and Fib-4 index. For example, for a patient with an albumin level of 38 g/L, bilirubin level of 18 μmol/L and Fib-4 index of 2, our RODIC model would predict a 5% risk of decompensation. In comparison, a patient whose albumin level is 30 g/L, bilirubin level is 35 μmol/L, and Fib-4 index is 4 would have a predicted decompensation risk of 39% (see Table S1, Supplementary Digital Content, http://links.lww.com/AJG/D916).

RODIC can be accessed free of charge online at: https://antonkaly.pythonanywhere.com/predict.

When assessing the performance of RODIC, we found that after 1,000 independent tests, average AUC-ROC and weighted F1 scores were 0.86 (95% CIs: 0.84–0.89) and 0.82 (95% CIs: 0.81–0.83), respectively (Figure 1a, Table 2), reflecting good stability across different sampling iterations and consistent effectiveness in distinguishing between predictor classes. When validated against external cohorts, the area under the curve (AUC) scores ranged from 0.67 to 0.80, with F1 scores ranging from 0.70 to 0.81 (Figure 1b, Table 2), further highlighting the great generalizability of our model. In addition, RODIC showed superior performance when compared with the individual RF analysis of additional liver function measures and scores in the same Data sets, including ALBI, AST to platelet ratio index, early prediction of decompensation score, the Fib-4 index, MELD and platet ALBI score (see Figure S1, Supplementary Digital Content, http://links.lww.com/AJG/D916).

**Figure 1.**
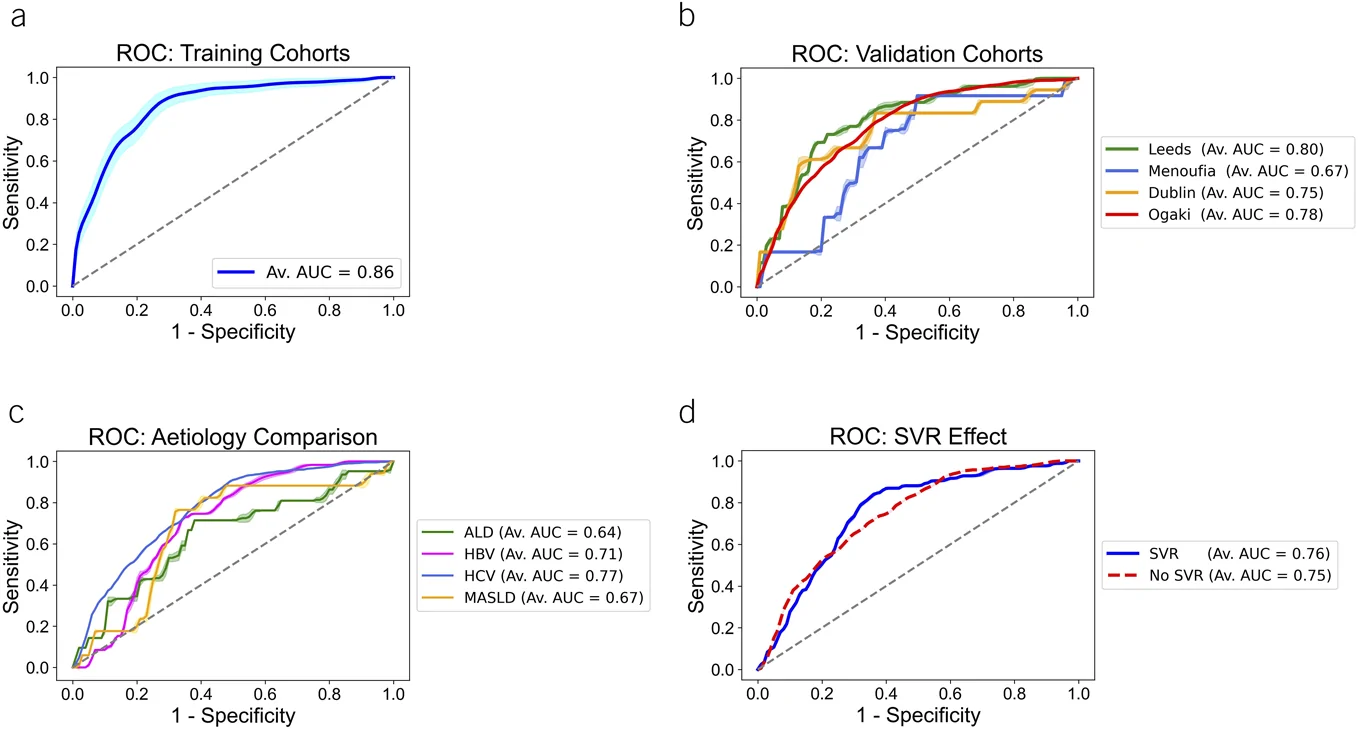
ROC curves showing the discriminatory performance of risk of decompensation in cirrhosis when (a) trained and tested in Nottingham (UK) and Modena (Italy) cohorts; (b) validated in Leeds (UK), Menoufia (Egypt), Dublin (Ireland), and Ogaki (Japan) cohorts; (c) validated in patients with ALD, HBV, HCV, and MASLD etiologies; (d) validated in patients with HCV with and without SVR. The mean AUC is presented with ± 1 SD around the mean. ALD, alcoholic liver disease; AUC, area under the curve; HBV, hepatitis B virus; HCV, hepatitis C virus; MASLD, metabolic dysfunction-associated steatotic liver disease; ROC, receiver operating characteristic; SVR, sustained virologic response.

**Table 2.**
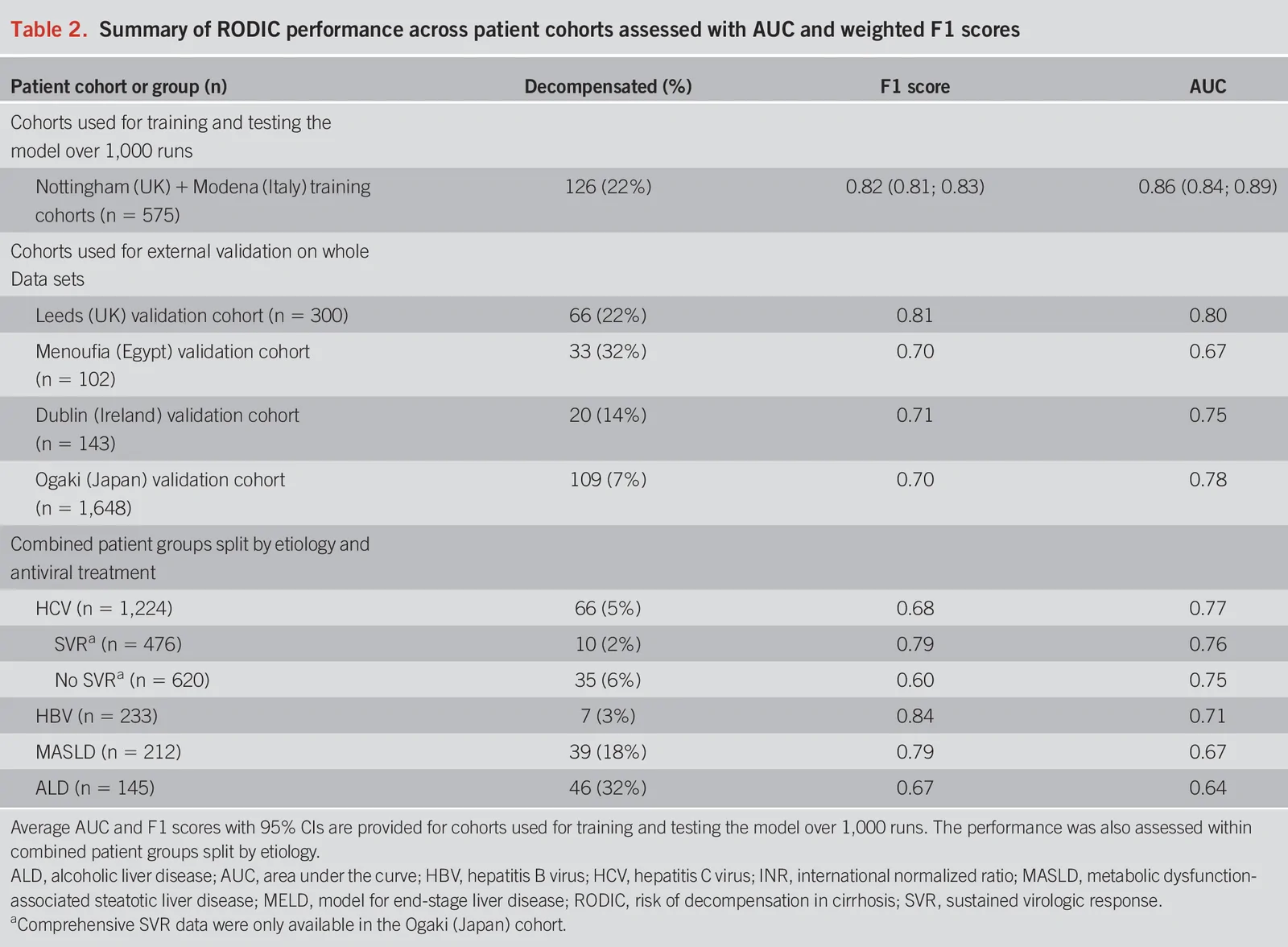
Summary of RODIC performance across patient cohorts assessed with AUC and weighted F1 scores

The model was calibrated using isotonic regression to adjust predicted probabilities so that they better reflected observed outcomes. Calibration performance was then evaluated (mean absolute error = 0.15; mean squared error = 0.05) and visualized with a standard calibration curve (Figure 2a).

**Figure 2.**
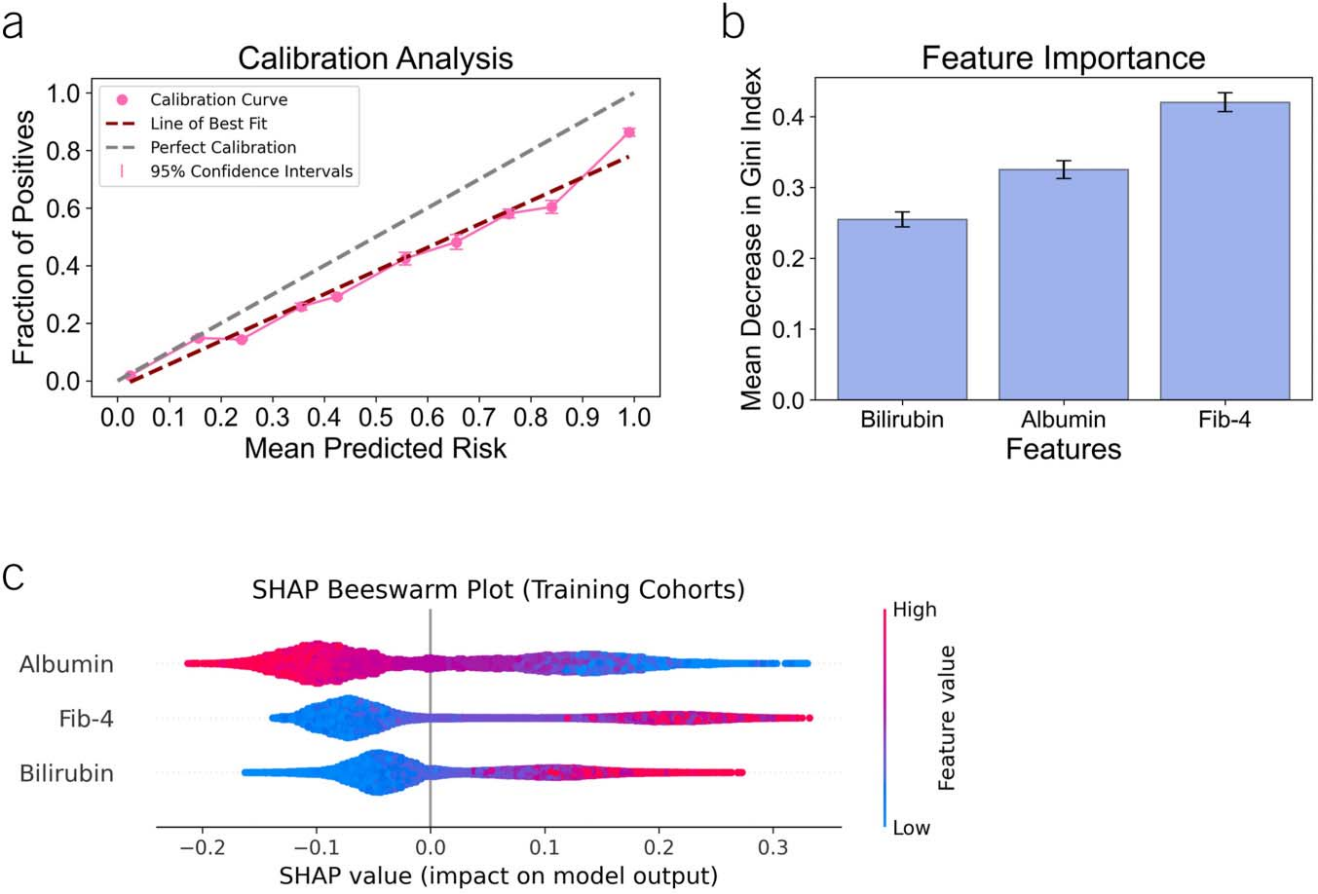
Calibration and feature assessment of RODIC. (a) Calibration curve of RODIC after 1,000 tests. The fraction of observed positive observations at each mean predicted risk interval is presented with 95% CIs. (b) The importance of patient features (variables) in RODIC presented as the relative mean decrease in Gini index per feature with standard error bars. (c) SHAP beeswarm plot of RODIC feature contributions aggregated across 100 tests of the training cohorts. The SHAP value corresponds to the direction of each feature's contribution to the predicted risk of decompensation, where positive SHAP values indicate increased predicted risk, whereas negative values indicate reduced risk. Color represents the feature value, with red indicating higher and blue indicating lower values. RODIC, risk of decompensation in cirrhosis; SHAP, shapley additive explanations.

### Patient variables

Of all selected variables used in RODIC, Fib-4 index had the highest relative importance when making predictions (Figure 2b). The RF performance was also higher when using the Fib-4 components as a composite index rather than incorporating them separately (see Figure S2, Supplementary Digital Content, http://links.lww.com/AJG/D916). A further SHAP analysis of patient features in both training (Figure 2c) and validation cohorts (see Figure S3, Supplementary Digital Content, http://links.lww.com/AJG/D916) demonstrated that the model predicts higher risk of decompensation for patients with high Fib-4, elevated bilirubin, and low albumin levels.

### Etiology analysis

In the etiology analysis, the alcoholic liver disease (ALD) group had the highest proportion of patients developing decompensation (32%), followed by metabolic dysfunction-associated steatotic liver disease (18%), hepatitis C virus (HCV) (5%) and hepatitis B virus (3%) (Table 2). Note that the number of ALD datapoints is relatively small and results should be treated with caution. RODIC performed generally well in all etiologies, with average AUC score ranging from 0.64 in the ALD group to 0.77 in the HCV group (Figure 1c; Table 2). In the HCV group from the Ogaki cohort (n = 1,096), 606 patients (55%) received antiviral treatment. Among them, 476 patients (79%) achieved sustained virologic response (SVR) and only 2% of those developed liver decompensation (Table 2). By contrast, among patients with HCV in the Ogaki cohort who either did not receive antiviral treatment (n = 490; 45%) or failed to achieve SVR (n = 130; 12%), 6% developed decompensation (Table 2). These patients were generally at a higher risk of decompensation compared with those who achieved SVR (log-rank test: *P* < 0.01) (Figure 3). RODIC maintained good predictive performance regardless of SVR status in patients with HCV (Figure 1d; Table 2).

**Figure 3.**
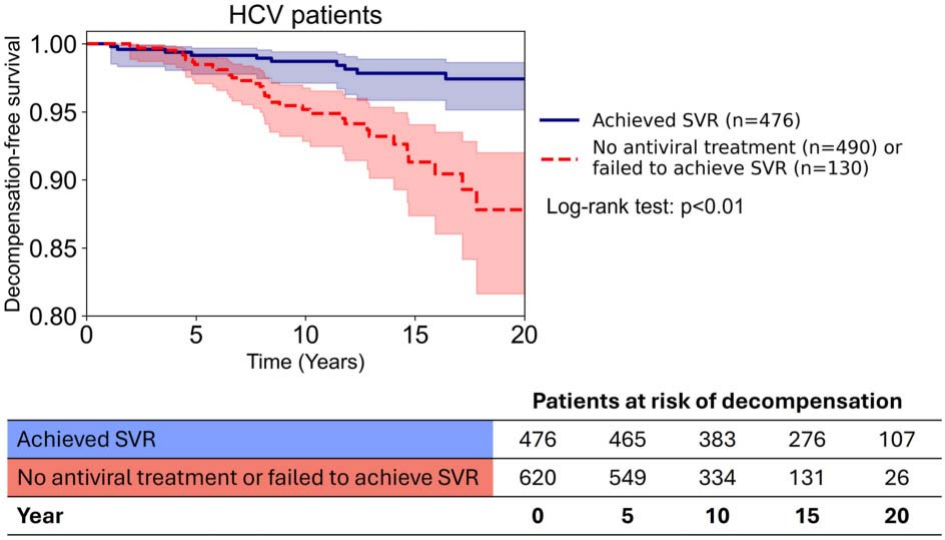
Estimated decompensation-free survival (with 95% CIs) in patients with HCV grouped by whether they achieved SVR (n = 476) or failed to achieve SVR either because of a failed antiviral treatment (n = 130) or having no antiviral treatment in the first place (n = 490). The results of the log-rank test indicate a significant difference in decompensation-free survival between the 2 groups. Patients at risk of decompensation are those who are still being followed up at a given timepoint and have not yet developed decompensation by that time. HCV, hepatitis C virus; SVR, sustained virologic response.

### Longitudinal analysis of RODIC and other risk scores

In the analysis of longitudinal changes of RODIC and MELD scores over time, patients who decompensated during follow-up had elevated decompensation risk 3 years before the event according to RODIC (Figure 4a). During that period, RODIC remained stable until around 6 months before decompensation at which point a more rapid rise was apparent. Similar findings were seen for Fib-4 (Figure 4b) and ALBI (Figure 4d). However, MELD showed no significant difference between groups until around 6 months before decompensation, as indicated by overlapping confidence intervals (Figure 4c).

**Figure 4.**
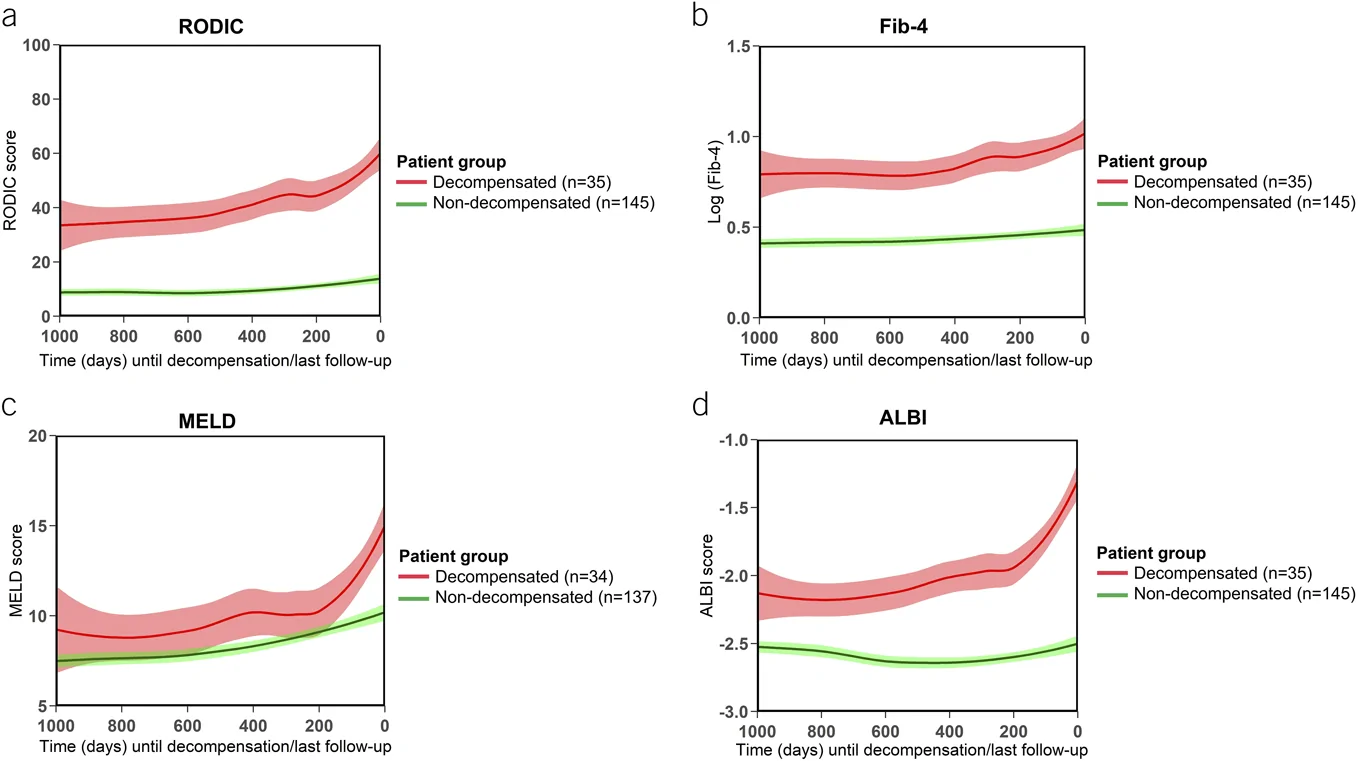
Longitudinal changes of (a) RODIC, (b) Fib-4, (c) MELD and (d) ALBI scores over time smoothed with locally estimated scatterplot smoothing regression with 95% CIs. Time 0 indicates the first point at which decompensation was evident or the time of last hospital check-up for patients who did not decompensate during follow-up. MELD, model for end-stage liver disease; RODIC, risk of decompensation in cirrhosis.

## DISCUSSION

Clinical decision algorithms are increasingly being introduced into medical practice (24). In our previous analysis, patients with compensated cirrhosis were only classified as being at high or low risk of decompensation (9), whereas with application of machine learning, more precise and quantitative estimates can be presented. Our new model numerically predicts the likelihood of decompensation occurring within the ensuing 3-year period. The high F1 and AUC-ROC scores (Table 2), alongside good calibration (Figure 2a), all attest to the model's discriminatory power, accuracy, and generalizability. However, we acknowledge that while all the cohorts were geographically distinct, we did not have details on their ethnic backgrounds and therefore cannot claim that our model is generalizable across different ethnicities.

RODIC was built entirely on the RF algorithm. The inherent randomness of the RF algorithm promotes diversity among the trees which ultimately reduces overfitting (11). The mechanism of making a prediction is also different from traditional regression methods which derive predictions from a specific mathematical equation based on input variables. Another key feature of the RF algorithm is its ability to estimate the importance of predictor variables. In this study, we used Gini-based feature importance, which quantifies the contribution of each variable to reducing node impurity across all trees. Furthermore, the RF algorithm treats all variables independently without assuming linearity, allowing for diverse, unstructured data features (11). Overall, the RF algorithm has been shown to have higher prediction accuracy compared with similar classifier models such as neural networks and boosting ensembles (25,26). Several studies have also demonstrated that the RF approach outperforms standard regression methods in outcome prediction across diverse Data sets (27–29) with its application also being highlighted in recent hepatology research (30–32).

RODIC has several advantages. First, it is built on 2 cohorts of patients followed up prospectively for several years with the specific purpose of documenting the natural history of compensated cirrhosis. Both training cohorts are characteristic of the experience of a Western center, whereas the Ogaki cohort, with the emphasis on chronic viral hepatitis, is more characteristic of Eastern experience. The diverse nature of our training cohorts enabled us to include multiple etiologies and assess SVR effects, supporting the model's performance across etiologies and treatments (Figure 1; Table 2).

Second, while the Nottingham, Modena and Ogaki cohorts are all prospectively accrued, the remaining Data sets were derived from routine clinical practice, countering the notion that clinical trial data are unrepresentative of routine real-world clinical scenarios. This is crucial for the practical implementation of any model. Third, the model uses only a small number of widely available and routinely measured clinical parameters. For all these reasons we believe that our model will be globally applicable to all common types/etiologies of cirrhosis. We envisage that RODIC would find application in the primary care setting where the detection of risk above a certain threshold would trigger referral for long term management. Our simple online calculator would greatly facilitate this application. In the secondary care setting, we suggest that the frequency of follow-up could be adapted to the risk of decompensation with the opportunity to better focus resources and make earlier interventions.

Although transient elastography (TE) has gained great attention for predicting hepatic decompensation in patients with compensated advanced chronic liver disease (33), our model may offer comparable prognostic performance at a much lesser cost. Given its noninvasive nature, accessibility and potential for integration into routine laboratory workflows, RODIC could complement or even rival TE in prognostic accuracy. Further comparative studies are warranted to validate their predictive power and define their role in risk stratification strategies alongside or in place of TE.

The major clinical consequence of cirrhosis is portal hypertension secondary to the associated fibrosis that is responsible for its most severe complications. Consistent with this is the observation that the most influential variable in our model is Fib-4 (Figure 2B), a well-validated index of hepatic fibrosis (34). SHAP analysis (Figure 2C) further highlights the importance of Fib-4, indicating that high Fib-4, as well as elevated bilirubin and low albumin values contribute to higher predicted risk of decompensation. It is important to emphasize that Fib-4 is not used in this study to assess hepatic fibrosis per se. Instead, it is included to provide the model with a more mechanistic and interpretable basis. Our RF analysis evaluates all components of Fib-4 and identifies each as relevant to the model, achieving the best performance when these components are combined. In the subsequent iterations of our model, we also anticipate being able to consider more direct measurements of portal hypertension such as HVPG and liver stiffness as estimated by TE (6,35). In the Italian Data set, HVPG has been measured and recorded, and its impact on decompensation will be reported separately (19).

It is not surprising that alternative measures of liver function (Fig. S1) also show some utility in the context of assessing liver failure. One of the striking examples is that the previously published EPOD model (36), developed using a completely different Data set, analytical approach, regional population, and methodology, identified the same prognostic factors, namely albumin, bilirubin, and platelets (1 of the Fib-4 components). These observations attest to the robustness of our model.

Consistent with the Baveno consensus (1), we have confined the term hepatic decompensation to ascites, encephalopathy, and variceal hemorrhage, omitting other parameters such as jaundice, hepatorenal syndrome, or HCC which have sometimes been considered as indicators of decompensation (37,38). There is also a move to distinguish different forms of decompensation based both on their time course and pathophysiology (39). Because no patients in our cohorts had recorded decompensation before study entry, they are classified as having stable compensated cirrhosis. Although the resulting raw decompensation rates differed between etiologies in our cohorts (e.g., higher proportions of ALD and metabolic dysfunction-associated steatotic liver disease patients developed decompensation compared with those with viral etiologies), our analysis (see Figure S2, Supplementary Digital Content, http://links.lww.com/AJG/D916) indicated that etiology contributed relatively little predictive value when examined alongside variables reflecting inflammation, fibrosis, and portal hypertension (40).

There are several intrinsic limitations to the performance of any model that aims to predict decompensation. Thus, the events that characterize decompensation are difficult to record in a consistent manner. For example, the presence or absence of ascites and HE relies on the sensitivity of methods applied (41). Similarly, some authorities consider jaundice to be 1 feature of decompensation but its clinical diagnosis either by visual inspection or by using an arbitrary serum bilirubin level can lead to inevitable interobserver inconsistencies. Ultrasound scanning will be more sensitive than clinical examination in the detection of ascites and European Association for the Study of the Liver guidelines note that there are no universally accepted standards for the definition, diagnosis, and classification of HE (42). Second, other variables that affect decompensation such as alcohol consumption and compliance with prescribed drugs such as beta blockers are difficult or impossible to document consistently or systematically. Third, although we may describe our cohorts as representing the natural history of compensated cirrhosis, it must be acknowledged that the changing therapeutic landscape will affect the natural history of the disease over time. Most directly, clinical interaction may reduce fibrosis or slow its progression, potentially lowering decompensation risk (43,44). Key developments include highly effective antiviral therapies for HCV (45), obesity management (46), and controlling portal hypertension (1). Our analysis confirms that achieving SVR in patients with chronic HCV infection does indeed decrease the risk of decompensation (Figure 3). However, the risk is not entirely eliminated, which could be a result of increased weight gain after antiviral treatment (47) or limited improvement in the underlying inflammatory milieu (48). Another factor which is likely to influence the rate of decompensation is the prevention of gastrointestinal varices which requires active screening and early therapeutic interventions (49). Finally, it is vital to consider HCC as a major outcome of compensated liver cirrhosis. Although this analysis predicts decompensation risk, future work will integrate the relationship between decompensation and HCC to develop a comprehensive model for predicting multiple cirrhosis outcomes.

## CONFLICTS OF INTEREST

**Guarantor of the article:** Philip J. Johnson, MD.

**Specific author contributions:** P.J.J.: study design, data collection and analysis, manuscript write up and review. A.K., A.G.: data analysis, manuscript write up and review. H.T.: data collection, manuscript write up and review. E.V.: data collection, data analysis, manuscript write up and review. O.E.: data collection, manuscript write up and review. S.S.: data collection, manuscript write up and review. I.A.R.: data collection, manuscript write up and review. J.S.: data collection. I.N.G.: study design, data collection, manuscript write up and review.

**Financial support:** Associazione Italiana sulla Ricerca sul Cancro (AIRC, grant no. IG 2020-ID, 24858 project awarded to E.V.).

**Potential competing interests:** None to report.

**Study Highlights**

**WHAT IS KNOWN**

✓ Hepatic decompensation is a major complication in patients with compensated liver cirrhosis.
✓ Predicting the risk of decompensation using simple and effective tools represents an unmet need.

**WHAT IS NEW HERE**

✓ Our new predictive model, RODIC, accurately quantitates the risk of developing decompensation within 3 years.
✓ RODIC demonstrates high predictive performance regardless of patient etiology and antiviral treatment.
✓ RODIC can help direct patient management strategies in clinical care and facilitate early interventions.

## Supplementary Material

## ABBREVIATIONS:

ALD: alcoholic liver disease
ALT: alanine transaminase
AST: aspartate aminotransferase
AUC: area under the curve
AUC-ROC: area under the receiver operating characteristic curve
CI: confidence interval
HBV: hepatitis B virus
HCC: hepatocellular carcinoma
HCV: hepatitis C virus
HE: hepatic encephalopathy
HVPG: hepatic vein portal gradient
MAE: mean absolute error
MASLD: Metabolic dysfunction-Associated Steatotic Liver Disease
MSE: mean squared error
RF: random forest
SVR: sustained virologic response
TE: transient elastography
