# Supplementary material for: Predicting the Risk of Decompensation in Patients With Cirrhosis: A Validated Machine Learning Approach
Source: Am J Gastroenterol. 2026 Mar 25;121(11):2659–68. doi: 10.14309/ajg.0000000000004004 (PMC13722424; doi:10.14309/ajg.0000000000004004)
Supplement: Supplementary file 1 [file acg-121-2659-s001.docx]

**Predicting the Risk of Decompensation in Patients with Cirrhosis – a Validated Machine Learning Approach**

**Supplementary Material**

**Authors**: Philip J Johnson^1^, Anton Kalyuzhnyy^2,3^, Arturas Grauslys^2,4^, Hidenori Toyoda^5^, Erica Villa^6^, Omar Elshaarawy^7^, Stephen Stewart^8^, Ian A Rowe^9^, Jessica Shearer^9^, Indra Neil Guha^10^.

1. Department of Molecular and Clinical Cancer Medicine, University of Liverpool, Liverpool, United Kingdom.
2. Computational Biology Facility, University of Liverpool, Liverpool, United Kingdom.
3. Hangzhou Zhisuan Anneng MedTech Ltd., Hangzhou, China.
4. Eliptica, London Cancer Hub, Sutton, London, United Kingdom.
5. Department of Gastroenterology, Ogaki Municipal Hospital, Ogaki, Japan.
6. CHIMOMO Department, University of Modena & Reggio Emilia, Modena, Italy.
7. Royal Liverpool and Broadgreen University Hospital NHS Trust, Liverpool, United Kingdom.
8. Department of Gastroenterology, The Mater Hospital, Dublin, Ireland.
9. Leeds Institute for Medical Research, University of Leeds, Leeds, United Kingdom & St James's University Hospital, Leeds, United Kingdom.
10. NIHR Nottingham Biomedical Research Centre, Nottingham University Hospitals NHS Trust and the University of Nottingham, Nottingham, United Kingdom.

**Corresponding author**: Prof. Philip J Johnson, MD.

Department of Molecular and Clinical Cancer Medicine, University of Liverpool, Liverpool, UK.

[Philip.Johnson@liverpool.ac.uk](mailto:Philip.Johnson@liverpool.ac.uk); ORCID number: 0000-0003-1404-0209; +44 (0)151 795 8410

| **Table of contents** | |
| --- | --- |
| **Table S1.** Examples of liver decompensation risk predictions made by RODIC | Page 1 |
| **Figure S1.** The relative importance of all patient features (variables) from the Nottingham cohort | Page 2 |
| **Figure S2.** The comparison of ROC curves between RODIC and other patient features | Page 3 |
|  |  |
| **Methods -** further details on model development, selecting patient features and model validation | Pages 4-5 |

**Supplementary Tables**

**Table S1**. Examples of liver decompensation risk predictions made by RODIC based on different patient values for albumin, bilirubin and Fib-4 index.

| **Patient example** | **Albumin (g/L)** | **Bilirubin (µmol/L)** | **Fib-4 index** | **Risk of decompensation within 3 years predicted by RODIC (%)** |
| --- | --- | --- | --- | --- |
| A | 38 | 18 | 2 | 5% |
| B | 30 | 35 | 4 | 39% |
| C | 27 | 50 | 17 | 90% |

**Supplementary Figures**

**Figure S1.** The importance of all patient features (variables) from the Nottingham cohort tested during the development of the random forest model, presented as relative mean decrease in Gini index per feature with standard error bars.

**Figure S2.** Receiver operating characteristic (ROC) curves showing the discriminatory performance of RODIC from the random forest analysis of the training cohorts (Nottingham and Modena) when compared to additional measures and scores including ALBI, APRI, EPOD, the Fib-4 index, MELD and PALBI.

**Methods – Further Details**

**Model development and internal validation**

All available complete patient measurements were split into positive and negative decompensation predictor classes. Measurements from non-decompensated patients were assigned to the negative class, while those from decompensated patients recorded within 3 years prior to decompensation were assigned to the positive class. Measurements recorded after decompensation or more than 3 years before decompensation were excluded.

To train and test the RF model, the data from the training cohorts was randomly split into training and testing sets at a patient level. In particular, 75% of randomly selected patients from each predictor class and all of their measurements went into the training set, whereas the measurements from the remaining 25% of patients were assigned to the testing set, ensuring sufficient data for training while retaining enough unseen data to reliably assess performance. Importantly, splitting the data at the patient level maintained the independence of the two sets and prevented data leakage, ultimately allowing to evaluate the model's performance on truly unseen patients. The process of random patient splitting was repeated across 1,000 iterations to estimate the model's generalisation across different patient groups^1^.

For each iteration, the RF model was trained and tested using Python (ver. 3.7.3) and open-source toolkit scikit-learn (ver. 1.0)^2^ with optimised hyperparameters determined via grid search. Specifically, the model used 1,000 trees (*n_estimators*) to provide robust internal validation and a maximum tree depth of 3 (*max_depth*) to limit overfitting while capturing key patterns in the data. To address class imbalance, cost-sensitive learning was applied by tuning the *class_weight* hyperparameter, resulting in an optimised ratio of 7.14:1 favouring the positive (decompensated) class.

**Selecting patient variables**

The model was first tested using all patient variables from the training cohorts that were deemed important for liver-related mortality/decompensation based on the authors' clinical experience, a review of relevant literature and our previous publication^3^. These included patient age, aetiology, platelet count, bilirubin, albumin, creatinine, sodium, aspartate aminotransferase (AST), alanine transaminase (ALT) and Fib-4 index^3-5^. All variables were assessed in terms of their influence on the model’s performance by measuring a total reduction of Gini index, where higher reduction implied higher importance. After extensive testing of different patient variables, as well as their combinations and possible composite scores such as Fib-4 index and ALBI score, a total of three most influential features (albumin, bilirubin and the Fib-4 index) were selected for the final model that, when combined, yielded the highest performance.

**External validation**

The RF model was validated on the four independent external cohorts (Table 1). To maximise the use of the training data and evaluate the performance of the model in as many unseen patients as possible, the model was trained on 100% of the training cohorts and then tested on all the complete biomarker measurements in each of the validation cohorts. For each validation cohort, AUC-ROC and F1 scores were collected.

The combined data from our validation cohorts enabled model assessment in separate aetiology populations, including hepatitis C virus (HCV), hepatitis B virus (HBV), metabolic dysfunction-associated steatotic liver disease (MASLD) and alcoholic liver disease (ALD). The Ogaki cohort contained comprehensive sustained virologic response (SVR) data in HCV patients which was used to test the model in patients with and without successful antiviral treatment.

**Decompensation-free survival of HCV patients**

The decompensation-free survival of HCV patients was estimated with Kaplan-Meier analysis and compared between SVR and no SVR groups using a log-rank statistical test^6^.

**References**

1.	Xu Q-S, Liang Y-Z. Monte Carlo cross validation. *Chemometrics and Intelligent Laboratory Systems*. 2001/04/16/ 2001;56(1):1-11. doi:<https://doi.org/10.1016/S0169-7439(00)00122-2>

2.	Pedregosa F, Varoquaux G, Gramfort A, et al. Scikit-learn: Machine Learning in Python. *J Mach Learn Res*. 2011;12:2825–2830.

3.	Guha IN, Harris R, Berhane S, et al. Validation of a Model for Identification of Patients With Compensated Cirrhosis at High Risk of Decompensation. *Clin Gastroenterol Hepatol*. Oct 2019;17(11):2330-2338 e1. doi:10.1016/j.cgh.2019.01.042

4.	Mallik M, Singhai A, Khadanga S, et al. The Significant Morbidity and Mortality Indicators in Patients of Cirrhosis. *Cureus*. Jan 2022;14(1):e21226. doi:10.7759/cureus.21226

5.	Roh YH, Kang BK, Jun DW, et al. Role of FIB-4 for reassessment of hepatic fibrosis burden in referral center. *Sci Rep*. Jun 30 2021;11(1):13616. doi:10.1038/s41598-021-93038-6

6.	Bland JM, Altman DG. The logrank test. *Bmj*. May 1 2004;328(7447):1073. doi:10.1136/bmj.328.7447.1073
